# Consensus transcriptional regulatory networks of coronavirus-infected human cells

**DOI:** 10.1038/s41597-020-00628-6

**Published:** 2020-09-22

**Authors:** Scott A. Ochsner, Rudolf T. Pillich, Neil J. McKenna

**Affiliations:** 1 The Signaling Pathways Project and Department of Molecular and Cellular Biology, Baylor College of Medicine, Houston, TX 77030 USA; 2 Department of Medicine, University of California San Diego, La Jolla, CA 92093 USA

**Keywords:** Cellular signalling networks, Viral host response, Data integration, Gene regulatory networks

## Abstract

Establishing consensus around the transcriptional interface between coronavirus (CoV) infection and human cellular signaling pathways can catalyze the development of novel anti-CoV therapeutics. Here, we used publicly archived transcriptomic datasets to compute consensus regulatory signatures, or consensomes, that rank human genes based on their rates of differential expression in MERS-CoV (MERS), SARS-CoV-1 (SARS1) and SARS-CoV-2 (SARS2)-infected cells. Validating the CoV consensomes, we show that high confidence transcriptional targets (HCTs) of MERS, SARS1 and SARS2 infection intersect with HCTs of signaling pathway nodes with known roles in CoV infection. Among a series of novel use cases, we gather evidence for hypotheses that SARS2 infection efficiently represses E2F family HCTs encoding key drivers of DNA replication and the cell cycle; that progesterone receptor signaling antagonizes SARS2-induced inflammatory signaling in the airway epithelium; and that SARS2 HCTs are enriched for genes involved in epithelial to mesenchymal transition. The CoV infection consensomes and HCT intersection analyses are freely accessible through the Signaling Pathways Project knowledgebase, and as Cytoscape-style networks in the Network Data Exchange repository.

## Introduction

Infection of humans by coronaviruses (CoV) represents a major current global public health concern. Signaling within and between airway epithelial and immune cells in response to infections by CoV and other viruses is coordinated by a complex network of signaling pathway nodes. These include chemokine and cytokine-activated receptors, signaling enzymes and transcription factors, and the transcriptional targets encoding their downstream effectors^1–3^. Placing the transcriptional events resulting from CoV infection in context with those associated with host signaling systems has the potential to catalyze the development of novel therapeutic approaches. The CoV research community has been active in generating and archiving transcriptomic datasets documenting the transcriptional response of human cells to infection by the three major CoV strains, namely, Middle East respiratory syndrome coronavirus (MERS-CoV, or MERS) and severe acute respiratory syndrome coronaviruses 1 (SARS-CoV-1, or SARS1) and 2 (SARS-CoV-2, or SARS2)^4–9^. To date however the field has lacked a resource that fully capitalizes on these datasets by, firstly, using them to identify human genes that are most consistently transcriptionally responsive to CoV infection and secondly, contextualizing these transcriptional responses by integrating them with ‘omics data points relevant to host cellular signaling pathways.

We recently described the Signaling Pathways Project (SPP)^10^, an integrated ‘omics knowledgebase designed to assist bench researchers in leveraging publically archived transcriptomic and ChIP-Seq datasets to generate research hypotheses. A unique aspect of SPP is its collection of consensus regulatory signatures, or consensomes, which rank genes based on the frequency of their significant differential expression across transcriptomic experiments mapped to a specific signaling pathway node or node family. By surveying across multiple independent datasets, we have shown that consensomes recapitulate regulatory relationships between signaling pathway nodes and their transcriptional targets to a high confidence level^10^. Here, as a service to the research community to catalyze the development of novel CoV therapeutics, we generated consensomes for infection of human cells by MERS, SARS1 and SARS2 CoVs. Computing the CoV consensomes against those for a broad range of cellular signaling pathway nodes, we discovered robust intersections between genes with high rankings in the CoV consensomes and those of nodes with known roles in the response to CoV infection. Integration of the CoV consensomes with the existing universes of SPP transcriptomic and ChIP-Seq data points in a series of use cases illuminates previously uncharacterized interfaces between CoV infection and human cellular signaling pathways. Moreover, while this paper was under review, numerous contemporaneous and independent *in vitro* and *in vivo* studies came to light that corroborate SARS2 network predictions made using our analysis pipeline. To reach the broadest possible audience of experimentalists, the results of our analysis were made available in the SPP website, as well as in the Network Data Exchange (NDEx) repository. Collectively, these networks constitute a unique and freely accessible framework within which to generate mechanistic hypotheses around the transcriptional interface between human signaling pathways and CoV infection.

## Results

### Generation of the CoV consensomes

We first set out to generate a set of consensomes^10^ ranking human genes based on statistical measures of the frequency of their significant differential expression in response to infection by MERS, SARS1 and SARS2 CoVs. To do this we searched the Gene Expression Omnibus (GEO) and ArrayExpress databases to identify datasets involving infection of human cells by these strains. Many of these datasets emerged from a broad-scale systematic multi-omics Pacific Northwest National Library analysis of the host cellular response to infection across a broad range of pathogens^11^. Since an important question in the development of CoV therapeutics is the extent to which CoVs have common transcriptional impacts on human cell signaling that are distinct from those of other viruses, we also searched for transcriptomic datasets involving infection by human influenza A virus (IAV). From this initial collection of datasets, we next carried out a three step quality control check as previously described^10^, yielding a total of 3.3 million data points in 156 experiments from 38 independent viral infection transcriptomic datasets (see figshare F1^12^, section 1). Using these curated datasets, we next used consensome analysis (see Methods and previous SPP publication^10^) to generate consensomes for each CoV strain. The full consensomes are available in figshare File F1^12^ sections 2 (SARS1), 3 (SARS2), 4 (MERS) and 5 (IAV). To assist researchers in inferring viral infection-associated signaling networks, the consensomes are annotated using the previously described SPP convention^10^ to indicate the identity of a gene as encoding a receptor, protein ligand, enzyme, transcription factor, ion channel or co-node (figshare File F1^12^, sections 2-5, columns A-C). In addition, to facilitate identification of transcripts that are selectively regulated in response to a specific CoV, or in response to CoV vs IAV infection, transcript percentile rankings for other consensomes are provided in each consensome tab in figshare FileF1^12^. Finally, Table 1 contains links to consensomes in the SPP knowledgebase and NDEx repository – see section “Visualization of the CoV transcriptional regulatory networks in the Signaling Pathways Project knowledgebase and Network Data Exchange repository” below and the instructional YouTube video (http://tiny.cc/2i56rz; strategies 2 (SPP) and 5 (NDEx)) for instructions on navigating these resources.

**Table 1:** DOI-driven links to consensomes and HCT intersection networks.

Virus	Resource	Network type	DOI	Ref.
MERS-CoV	SPP	Consensome	10.1621/jgxM527b8s.1	^129^
	NDEx	Consensome	10.18119/N9QG7S	^133^
		Node family HCT intersection	10.18119/N9PG63	^137^
		Node HCT intersection	10.18119/N96G6R	^141^
SARS-CoV-1	SPP	Consensome	10.1621/k9ygy4i49j.1	^130^
	NDEx	Consensome	10.18119/N9KP4G	^134^
		Node family HCT intersection	10.18119/N9JS46	^138^
		Node HCT intersection	10.18119/N92P56	^142^
SARS-CoV-2	SPP	Consensome	10.1621/vTiy8d4Iq7.1	^131^
	NDEx	Consensome	10.18119/N9G02W	^135^
		Node family HCT intersection	10.18119/N9F016	^139^
		Node HCT intersection	10.18119/N9Z01V	^143^
IAV	SPP	Consensome	10.1621/58AOyXDIAH.1	^132^
	NDEx	Consensome	10.18119/N9B60Z	^136^
		Node family HCT intersection	10.18119/N9989R	^140^
		Node HCT intersection	10.18119/N9T609	^144^

**SPP DOIs** point to the tabular web version of the consensome, which can be downloaded as an Excel file. **NDEx consensome DOIs** point to the full consensome network; for ease and clarity of display, only the top 5% of the consensome is shown in the initial graphic display; in addition, a subset of the data corresponding only to the top 5% of the consensome can be reached via a link in the “Description”. **NDEx virus node family HCT intersection DOIs** point to networks containing all node families; **NDEx virus node HCT intersection DOIs** point to the full HCT intersection network; for ease and clarity of display, only the top 5% of the HCT intersection network is shown in the initial graphic display; in addition, a subset of the data corresponding only to the top 5% of the HCT intersection network can be reached via a link in the “Description”.

### Ranking of interferon-stimulated genes (ISGs) in the CoV consensomes

**Fig. 1:**
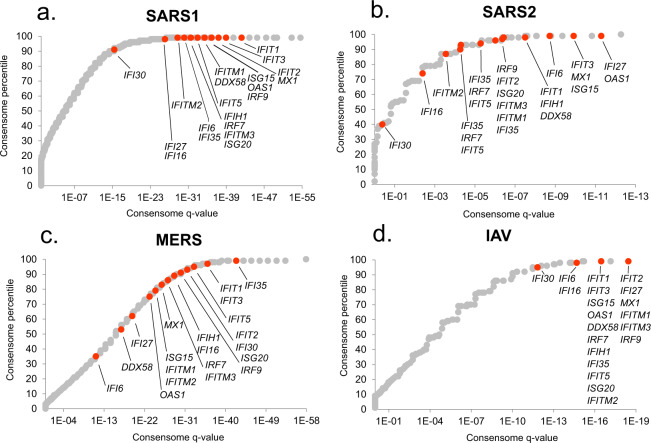
Rankings of canonical interferon-stimulated genes (ISGs) in the viral consensomes. Shown are the percentile rankings of 20 ISGS^13^ in the SARS1 **(a)**, SARS2 **(b)**, MERS **(c)** and IAV **(d)** consensomes. Note that numerous genes have identical *q*-value and percentile values and are therefore superimposed in the plots. Full consensome data are provided in figshare File 1^12^ sections 2 (SARS1), 3 (SARS2), 4 (MERS) and 5 (IAV); see also Table 1 for links to consensomes in the SPP knowledgebase and NDEx repository. Please refer to the Methods section for a full description of the consensome algorithm.

As an initial benchmark for our CoV consensome analysis, we assembled a list of 20 canonical interferon-stimulated genes (ISGs), whose role in the anti-viral response is best characterized in the context of IAV infection^13^. As shown in Fig. 1, many ISGs were assigned elevated rankings across the four viral consensomes. The mean percentile of the ISGs was however appreciably higher in the IAV (98.7^th^ percentile) and SARS1 (98.5^th^ percentile; *p* = 6e-1, t-test IAV vs SARS1) consensomes than in the SARS2 (92^nd^ percentile, *p* = 5e-2, t-test IAV v SARS2) and MERS (82^nd^ percentile; *p* = 7e-5, t-test IAV v MERS) consensomes. This is consistent with previous reports of an appreciable divergence between IAV and SARS2 infection with respect to the interferon transcriptional response^8^. Other genes with known critical roles in the response to viral infection have high rankings in the CoV consensomes, including *NCOA7*^14^ (percentiles: SARS1, 98^th^; SARS2, 97^th^; MERS, 89^th^; IAV, 99^th^), *STAT1*^15^ (percentiles: SARS1, 99^th^; SARS2, 98^th^; MERS, 89^th^; IAV, 99^th^) and *TAP1*^16^ (percentiles: SARS1, 99^th^; SARS2, 94^th^; MERS, 83^rd^; IAV, 99^th^). In addition to the appropriate elevated rankings for these known viral response effectors, the CoV consensomes assign similarly elevated rankings to transcripts that are largely or completely uncharacterized in the context of viral infection. Examples of such genes include *PSMB9*, encoding a proteasome 20S subunit (percentiles: SARS1, 98^th^; SARS2, 97^th^; MERS, 98^th^; IAV, 98^th^); *CSRNP1*, encoding a cysteine and serine rich nuclear protein (percentiles: SARS1, 99^th^; SARS2, 94^th^; MERS, 98^th^; IAV, 94^th^); and *CCNL1*, encoding a member of the cell cycle-regulatory cyclin family (percentiles: SARS1, 99^th^; SARS2, 94^th^; MERS, 99^th^; IAV, 97^th^). Finally, a preprint of a CRISPR/Cas9 study described validation of 27 human genes as critical modulators of the host response to SARS2 infection of human cells^17^. Corroborating our analysis, 16 of these genes have significant (*q* < 0.05) rankings in the SARS2 consensome, including *ACE2* and *DYRK1A* (both 97^th^ percentile), *CTSL* (96^th^ percentile), *KDM6A*, *ATRX*, *PIAS1* (all 94^th^ percentile), *RAD54L2* and *SMAD3* (90^th^ percentile).

To gather evidence for human signaling pathway nodes orchestrating the transcriptional response to CoV infection, we next compared transcripts with elevated rankings in the CoV consensomes with those that have predicted high confidence regulatory relationships with cellular signaling pathway nodes. We generated four lists of genes corresponding to the MERS, SARS1, SARS2 and IAV transcriptomic consensome 95^th^ percentiles. We then retrieved genes in the 95^th^ percentiles of available SPP human transcriptomic (n = 25) consensomes and ChIP-Seq (n = 864) pathway node consensomes^10^. For convenience we will refer from hereon to genes in the 95^th^ percentile of a viral infection, node (ChIP-Seq) or node family (transcriptomic) consensome as high confidence transcriptional targets (HCTs). We then used the R GeneOverlap package^18^ to compute the extent and significance of intersections between CoV HCTs and those of the pathway nodes or node families. We interpreted the extent and significance of intersections between HCTs for CoVs and pathway node or node families as evidence for a biological relationship between loss or gain of function of that node (or node family) and the transcriptional response to infection by a specific virus.

**Fig. 2:**
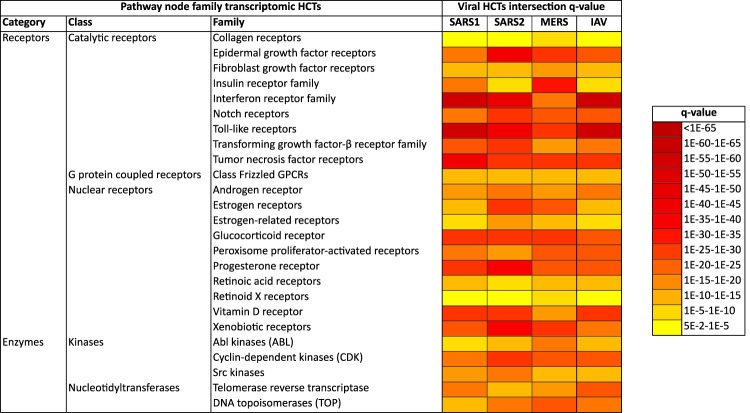
High confidence transcriptional target (HCT) intersection analysis of viral infection and human receptors or signaling enzymes based on transcriptomic consensomes. Due to space constraints some class and family names may differ slightly from those in the SPP knowledgebase. All *q*-values refer to those obtained using the GeneOverlap analysis package in R^18^. Full numerical data are provided in figshare File F1^12^, section 6; see also Table 1 for links to virus-node family HCT intersection networks in the NDEx repository.

**Fig. 3:**
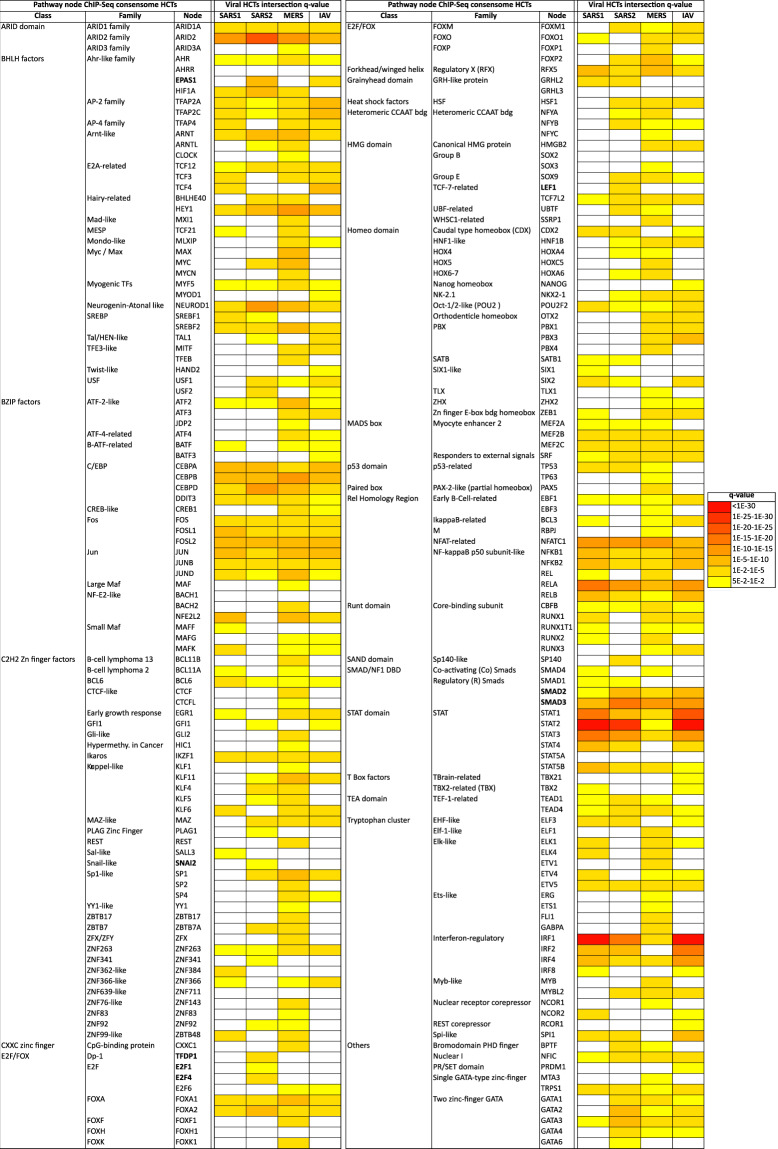
High confidence transcriptional target (HCT) intersection analysis of viral infection and human transcription factors based on ChIP-Seq consensomes. White cells represent *q* > 5e-2 intersections. Due to space constraints some class and family names may differ slightly from those in the SPP knowledgebase. All *q*-values refer to those obtained using the GeneOverlap analysis package in R^18^. Full numerical data are provided in figshare File F1^12^, section 7; see also Table 1 for links to virus-node HCT intersection networks in the NDEx repository.

**Fig. 4:**
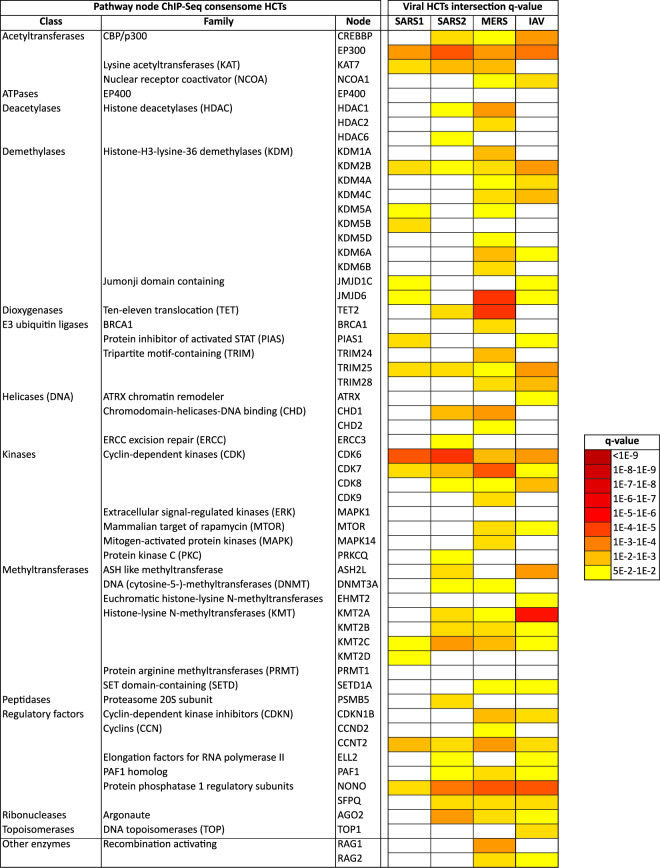
High confidence transcriptional target (HCT) intersection analysis of viral infection and human signaling enzymes based on ChIP-Seq consensomes. White cells represent non-significant (*q* > 5e-2) intersections. Due to space constraints some class and family names may differ slightly from those in the SPP knowledgebase. All *q*-values refer to those obtained using the GeneOverlap analysis package in R^18^. Full numerical data are provided in figshare File F1^12^, section 7; see also Table 1 for links to virus-node HCT intersection networks in the NDEx repository.

Results of viral infection and signaling node HCT intersection analyses are shown in Fig. 2 (based on receptor and enzyme family transcriptomic consensomes), Figs. 3 and 4 (based on ChIP-Seq consensomes for transcription factors and enzymes, respectively) and figshare File F2^12^ (based on ChIP-Seq consensomes for selected co-nodes). figshare File F1^12^, sections 6 (node family transcriptomic HCT intersection analysis) and 7 (node ChIP-Seq HCT intersection analysis) contain the full underlying numerical data. Please refer also to Table 1 for links to virus-node family and virus-node HCT intersection networks in the NDEx repository – see section “Visualization of the CoV transcriptional regulatory networks in the Signaling Pathways Project knowledgebase and Network Data Exchange repository” below and the instructional YouTube video (http://tiny.cc/2i56rz; strategy 6) for instructions on navigating these resources. We surveyed *q* < 0.05 HCT intersections to identify (i) canonical inflammatory signaling pathway nodes with characterized roles in the response to CoV infection, thereby validating the consensome approach in this context; and (ii) evidence for nodes whose role in the transcriptional biology of CoV infection is previously uncharacterized, but consistent with their roles in the response to other viral infections. In the following sections all *q*-values refer to those obtained using the GeneOverlap analysis package in R^18^.

**Receptors** Reflecting their well-documented roles in the response to CoV infection^19–22^, we observed appreciable significant intersections between CoV HCTs and those of the toll-like (TLRs; *q*-values: SARS1, 3e-85; SARS2, 5e-49; MERS, 2e-33), interferon (IFNR; *q*-values: SARS1, 1e-109; SARS2, 6e-53; MERS, 1e-24) and tumor necrosis factor (TNFR; *q*-values: SARS1, 1e-48; SARS2, 1e-35; MERS, 5e-32) receptor families (Fig. 2). Indeed, anti-TNF therapy has been recently mooted as a potential clinical approach to SARS2 infection^23^. HCT intersections between CoV infection and receptor systems with previously uncharacterized connections to CoV infection, including epidermal growth factor receptors (EGFR; *q*-values: SARS1, 4e-21; SARS2, 3e-48; MERS, 1e-35), and Notch receptor signaling (*q*-values: SARS1, 6e-24; SARS2, 2e-33; MERS, 2e-29; Fig. 2), are consistent with their known role in the context of other viral infections^24–28^. The Notch receptor HCT intersection points to a possible mechanistic basis for the potential of Notch pathway modulation in the treatment of SARS2^29^. The strong HCT intersection between CoV infection and xenobiotic receptors (*q*-values: SARS1, 1e-30; SARS2, 1e-44; MERS, 5e-32; Fig. 2) reflects work describing a role for pregnane X receptor in innate immunity^30^ and points to a potential role for members of this family in the response to CoV infection. In addition, the robust intersection between HCTs for SARS2 infection and vitamin D receptor (*q* = 2e-35) is interesting in light of epidemiological studies suggesting a link between risk of SARS2 infection and vitamin D deficiency^31,32^. Consistent with a robust signature for the glucocorticoid receptor across all CoVs (GR; *q*-values: SARS1, 3e-35; SARS2, 1e-35; MERS, 7e-32), recent studies have shown the GR agonist dexamethasone is a successful therapeutic for SARS2 infection^33^. Finally, independent *in vitro* analyses confirm our predictions of the modulation by SARS2 infection of ErbB/EGFR^22,34^ and TGFBR^17,34^ signaling systems (Fig. 2).

**Transcription factors** Not unexpectedly – and speaking again to validation of the consensomes - the strongest and most significant CoV HCT intersections were observed for HCTs for known transcription factor mediators of the transcriptional response to CoV infection, including members of the NFκB (*q*-value ranges: SARS1, 1e-7-1e-9; SARS2, 9e-3-2e-3; MERS, 1e-3-1e-4)^35–37^, IRF (*q*-value ranges: SARS1, 2e-2-1e-31; SARS2, 2e-4-1e-17; MERS, 9e-4-7e-5)^38^ and STAT (*q*-value ranges: SARS1, 1e-7-1e-55; SARS2, 2e-3-3e-29; MERS, 5e-2-3e-5)^39–41^ transcription factor families (Fig. 3). Consistent with the similarity between SARS1 and IAV consensomes with respect to elevated rankings of ISGs (Fig. 2a,d), the IRF1 HCT intersection was strongest with the SARS1 (*q* = 2e-34) and IAV (*q* = 3e-49) HCTs. Corroborating our finding of a strong intersection between STAT2 and SARS2 infection HCTs (*q = *3e-29), a recent preprint has shown that STAT2 plays a prominent role in the response to SARS2 infection of Syrian hamsters^42^. HCT intersections for nodes originally characterized as having a general role in RNA Pol II transcription, including TBP (*q*-values: SARS1, 2e-10; SARS2, 6e-23; MERS, 3e-16), GTF2B/TFIIB (*q*-values: SARS1, 7e-10; SARS2, 3e-23; MERS, 9e-14) and GTF2F1 (*q*-values: SARS1, 2e-4; SARS2, 2e-13; MERS, 5e-5) were strong across all CoVs, and particularly noteworthy in the case of SARS2. In the case of GTF2B, these data are consistent with previous evidence identifying it as a specific target for orthomyxovirus^43^, and the herpes simplex^44^ and hepatitis B^45^ viruses. Moreover, a recent preprint has identified a high confidence interaction between GTF2F2 and the SARS2 NSP9 replicase^34^.

In general, intersections between viral infection and ChIP-Seq enrichments for transcription factors and other nodes were more specific for individual CoV infection HCTs (compare Fig. 2 with Figs. 3 and 4 and figshare File F1^12^, sections 6 and 7). This is likely due to the fact that ChIP-Seq consensomes are based on direct promoter binding by a specific node antigen, whereas transcriptomic consensomes encompass both direct and indirect targets of specific receptor and enzyme node families.

**Enzymes** Compared to the roles of receptors and transcription factors in the response to viral infection, the roles of signaling enzymes are less well illuminated – indeed, in the context of CoV infection, they are entirely unstudied. Through their regulation of cell cycle transitions, cyclin-dependent kinases (CDKs) play important roles in the orchestration of DNA replication and cell division, processes that are critical in the viral life cycle. CDK6, which has been suggested to be a critical G1 phase kinase^46,47^, has been shown to be targeted by a number of viral infections, including Kaposi’s sarcoma-associated herpesvirus^48^ and HIV-1^49^. Consistent with this common role across distinct viral infections, we observed robust intersection between the CDK family HCTs (*q*-values: SARS1, 8e-23; SARS2, 2e-31; MERS, 1e-30; Fig. 2) and the CDK6 HCTs (*q*-values: SARS1, 1e-7; SARS2, 8e-8; MERS, 3e-4; Fig. 4) and those of all viral HCTs. As with the TLRs, IFNRs and TNFRs, which are known to signal through CDK6^50,51^, intersection with the CDK6 HCTs was particularly strong in the case of the SARS2 HCTs (Fig. 4). Again, the subsequent proteomic analysis we alluded to earlier^34^ independently corroborated our prediction of a role for CDK6 in the response to SARS2 infection. Consistent with a recent study^52^, the intersection of HCTs for the lysine demethylase KMT2A was much stronger with IAV (*q*-value = 2e-9) than with any of the CoVs (*q*-values: SARS1, 7e-2; SARS2, 2e-3; MERS, 1e-2).

CCNT2 is another member of the cyclin family that, along with CDK9, is a component of the viral-targeted p-TEFB complex^53^. Reflecting a potential general role in viral infection, appreciable intersections were observed between the CCNT2 HCTs and all viral HCTs (*q*-values: SARS1, 4e-4; SARS2, 6e-3; MERS, 7e-5; Fig. 4). Finally in the context of enzymes, the DNA topoisomerases have been shown to be required for efficient replication of simian virus 40^54^ and Ebola^55^ viruses. The prominent intersections between DNA topoisomerase-dependent HCTs and the CoV HCTs (*q*-values: SARS1, 3e-15; SARS2, 6e-21; MERS, 1e-26; Fig. 2) suggest that it may play a similar role in facilitating the replication of these CoVs.

### Hypothesis generation use cases

We next wished to show how the CoV consensomes and HCT intersection networks, supported by existing canonical literature knowledge, enable the user to generate novel hypotheses around the transcriptional interface between CoV infection and human cellular signaling pathways. Given the current interest in SARS2, we have focused our use cases on that virus. figshare File F2^12^ contains an additional use case linking the telomerase catalytic subunit TERT to CoV infection that was omitted from the main text due to space constraints. Unless otherwise stated, all *q*-values below were obtained using the GeneOverlap analysis package in R^18^. We stress that all use cases represent preliminary *in silico* evidence only, and require rigorous pressure-testing at the bench for full validation.

### Hypothesis generation use case 1: transcriptional regulation of the SARS2 receptor gene, *ACE2*

**Fig. 5:**
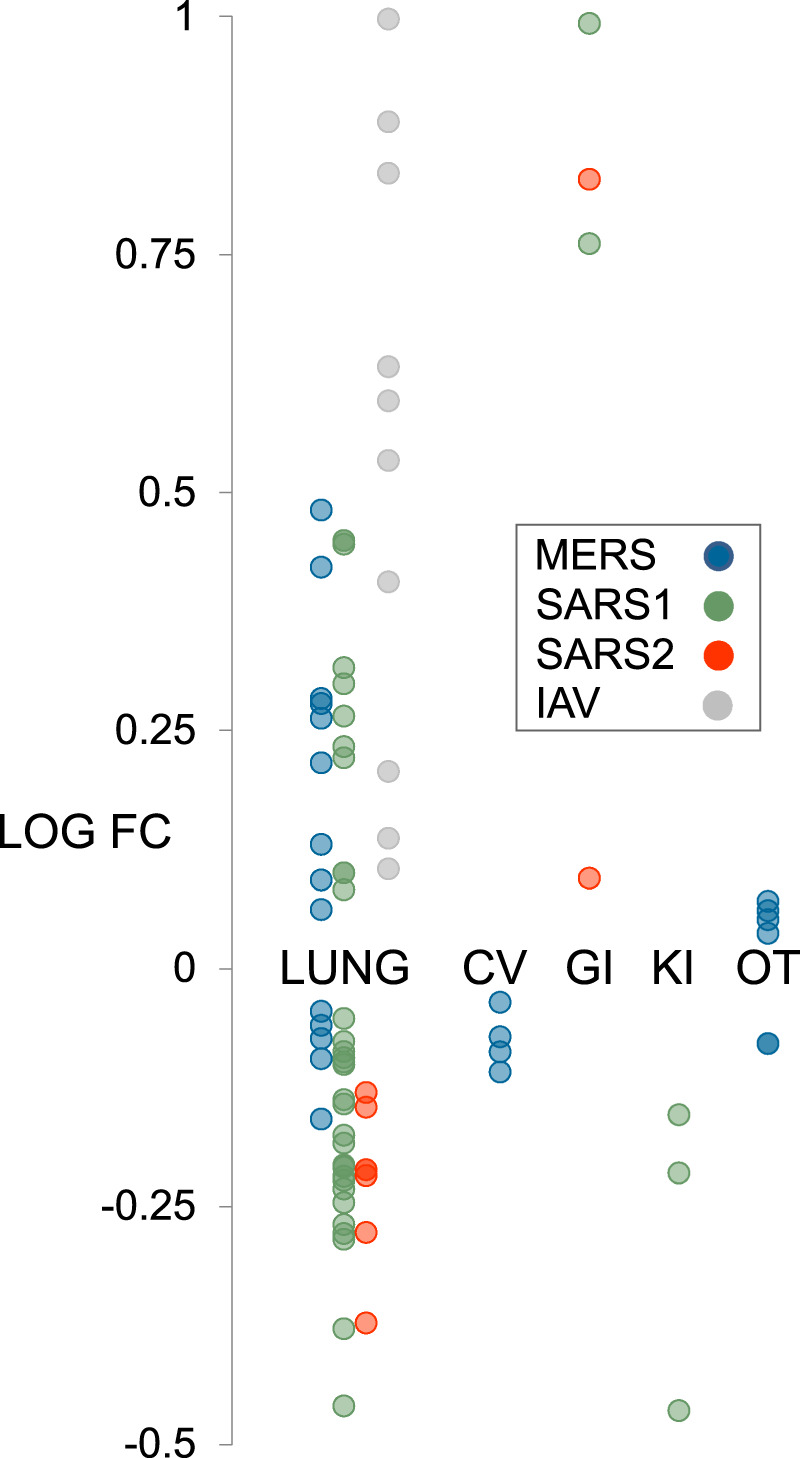
Hypothesis generation use case 1: strain- and tissue-specific regulation of *ACE2* in response to CoV infection of human cells. All data points are p < 0.05. Refer to figshare File F1^12^, section 1 for full details on the underlying datasets. Abbreviations: CV, cardiovascular; GI, gastrointestinal; KI, kidney; OT, others.

*ACE2*, encoding membrane-bound angiotensin converting enzyme 2, has gained prominence as the target for cellular entry by SARS1^56^ and SARS2^57^. An important component in the development of ACE2-centric therapeutic responses is an understanding of its transcriptional responsiveness to CoV infection. Interestingly, based on our CoV consensome analysis, *ACE2* is more consistently transcriptionally responsive to infection by SARS CoVs (SARS1: 98^th^ percentile, consensome *q* value (CQV)^10^ = 1e-25; SARS2: 97^th^ percentile, CQV = 4e-7) than by IAV (78^th^ percentile, CQV* = *3e-8) or MERS (49^th^ percentile, CQV* = *2e-16; figshare File F1^12^, sections 2-5). The data points underlying the CoV consensomes indicate evidence for tissue-specific differences in the nature of the regulatory relationship between *ACE2* and viral infection. In response to SARS1 infection, for example, *ACE2* is induced in pulmonary cells but repressed in kidney cells (Fig. 5). On the other hand, in response to SARS2 infection, *ACE2* is repressed in pulmonary cells - a finding corroborated by other studies^58,59^ - but inducible in gastrointestinal cells (Fig. 5). These data may relate to the selective transcriptional response of *ACE2* to signaling by IFNRs (92^nd^ percentile; figshare File F1^12^, section 8) rather than TLRs (48^th^ percentile; figshare File F1^12^, section 9) or TNFRs (13^th^ percentile, figshare File F1^12^, section 10). These findings are consistent with a recent study confirming repression of induction of *ACE2* by interferon stimulation and by IAV infection^60^. Our data reflect a complex transcriptional relationship between *ACE2* and viral infection that may be illuminated in part by future single cell RNA-Seq analysis in the context of clinical or animal models of SARS2 infection.

### Hypothesis generation use case 2: evidence for antagonistic cross-talk between progesterone receptor and interferon receptor signaling in the airway epithelium

**Fig. 6:**
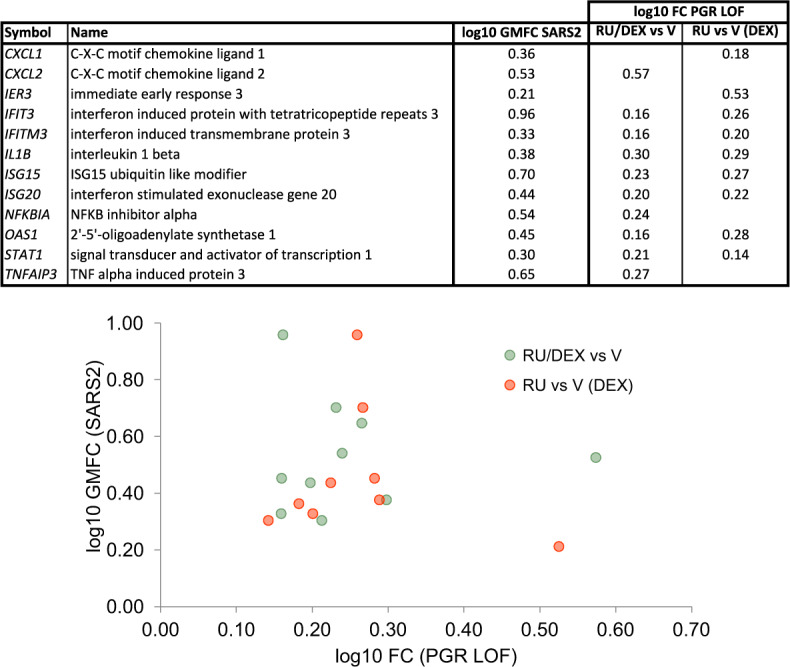
Hypothesis generation use case 2: antagonism between PGR and SARS2 inflammatory signaling in the regulation of viral response genes in the airway epithelium. GMFC: geometric mean fold change. PGR loss of function (LOF) experiments were retrieved from the SPP knowledgebase^145^.

A lack of clinical data has so far prevented a definitive evaluation of the connection between pregnancy and susceptibility to SARS2 infection in CoVID-19. That said, SARS2 infection is associated with an increased incidence of pre-term deliveries^61^, and pregnancy has been previously associated with the incidence of viral infectious diseases, particularly respiratory infections^62,63^. We were therefore interested to observe consistent intersections between the progesterone receptor (PGR) HCTs and CoV infection HCTs (*q*-values: SARS1, 3e-35; SARS2, 5e-41; MERS 5e-28), with the intersection being particularly evident in the case of the SARS2 HCTs (Fig. 2; figshare File F1^12^, section 6). To investigate the specific nature of the crosstalk implied by this transcriptional intersection in the context of the airway epithelium, we first identified a set of 12 genes that were HCTs for both SARS2 infection and PGR. Interestingly, many of these genes encode members of the classic interferon-stimulated gene (ISG) response pathway^13^. We then retrieved two SPP experiments involving treatment of A549 airway epithelial cells with the PGR full antagonist RU486 (RU), alone or in combination with the GR agonist dexamethasone (DEX). As shown in Fig. 6, there was unanimous correlation in the direction of regulation of all 12 genes in response to CoV infection and PGR loss of function. These data are consistent with the reported pro-inflammatory effects of RU486 in a mouse model of allergic pulmonary inflammation^64^. Interestingly, SARS2-infected pregnant women are often asymptomatic^65,66^. Based on our data, it can be reasonably hypothesized that suppression of the interferon response to SARS2 infection by elevated circulating progesterone during pregnancy may contribute to the asymptomatic clinical course. Indeed, crosstalk between progesterone and inflammatory signaling is well characterized in the reproductive system, most notably in the establishment of uterine receptivity^67^ as well as in ovulation^68^. Consistent with our hypothesis, a recently launched clinical trial is evaluating the potential of progesterone for treatment of COVID-19 in hospitalized men^69^. Interestingly, the recently reported inhibition by progesterone of SARS2 replication in Vero 6 cells^70^ indicates an additional mechanism, distinct from its potential crosstalk with the interferon response, by which progesterone signaling may impact SARS2 infection.

### Hypothesis generation use case 3: association of an epithelial to mesenchymal transition transcriptional signature with SARS2 infection

Epithelial to mesenchymal transition (EMT) is the process by which epithelial cells lose their polarity and adhesive properties and acquire the migratory and invasive characteristics of mesenchymal stem cells^71^. EMT is known to contribute to pulmonary fibrosis^72^, acute interstitial pneumonia^73^ and acute respiratory distress syndrome (ARDs)^74^, all of which have been reported in connection with SARS2 infection in COVID-19^75–77^. We were interested to note therefore that significant HCT intersections for three well characterized EMT-promoting transcription factors were specific to SARS2 infection (*q*-values: SNAI2/Slug^78^, 2e-2; EPAS1/HIF2α^79^, 9e-9; LEF1^80^, 1e-3; Fig. 3, bold symbols; figshare File F1^12^, section 7). Consistent with this, intersections between HCTs for TGFBRs, SMAD2 and SMAD3, known regulators of EMT transcriptional programs^81^ – were stronger with HCTs for SARS2 (*q*-values: TGFBRs, 2e-31; SMAD2, 2e-7; SMAD3, 5e-17) than with those of SARS1 (*q*-values: TGFBRs, 6e-29; SMAD2, 2e-2; SMAD3, 3e-9) and MERS (*q*-values: TGFBRs, 1e-16; SMAD2, 3e-3; SMAD3, 2e-12) – see also Figs. 2 and 3 and figshare File F1^12^, sections 6 and 7). Moreover, a recent CRISPR/Cas9 screen identified a requirement for both TGFBR signaling and *SMAD3* in mediating SARS2 infection^17^.

**Fig. 7:**
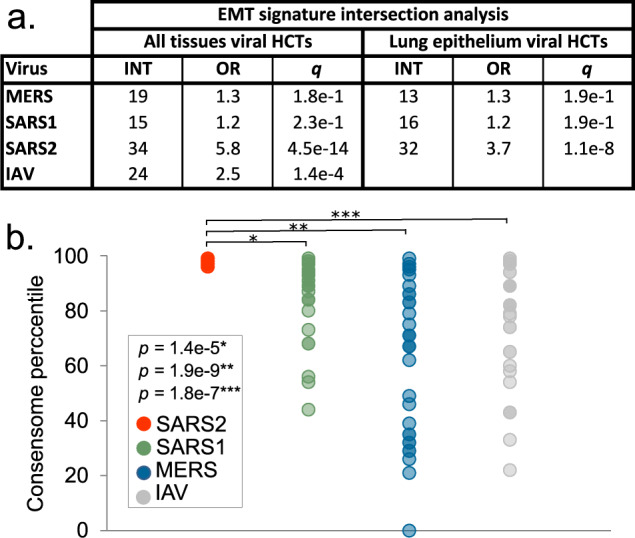
Hypothesis generation use case 3: evidence for a SARS2 infection-associated EMT transcriptional signature. (**a**) CoV HCT intersection (INT) with the literature-curated EMT signature for all-biosample and lung epithelium-specific consensomes. The IAV consensome is comprised of lung epithelial cell lines and was therefore omitted from the lung epithelium-only consensome analysis. Refer to the column “EMT” in figshare File F1^12^, section 3 for the list of EMT SARS2 HCTs. *q*-values refer to those obtained using the GeneOverlap analysis package in R^18^. (**b**) Comparison of mean percentile ranking of the EMT-associated SARS2 HCTs across viral consensomes. Note that SARS2 HCTs are all in the 97–99^th^ percentile and are therefore superimposed in the scatterplot. Indicated are the results of the two-tailed two sample t-test assuming equal variance comparing the percentile rankings of the SARS2 EMT HCTs across the four viral consensomes.

To investigate the connection between SARS2 infection and EMT implied by these HCT intersections, we then computed intersections between the individual viral HCTs and a list of 335 genes manually curated from the research literature as EMT markers^82^, see also figshare File F1^12^, section 11. In agreement with the HCT intersection analysis, we observed significant enrichment of members of this gene set within the SARS2 HCTs (*q = *4e-14), but not the SARS1 or MERS (both *q = *2e-1) HCTs (Fig. 7a). Consistent with previous reports of a potential link between EMT and IAV infection^83^, we observed significant intersection between the EMT signature and the IAV HCTs (*q = *1e-4).

One possible explanation for the selective intersection between the literature EMT signature and the SARS2 HCTs relative to SARS1 and MERS was the fact that the SARS2 consensome was exclusively comprised of epithelial cell lines, whereas the SARS1 and MERS consensomes included non-epithelial cell biosamples (figshare File F1^12^, section 1). To exclude this possibility therefore, we next calculated airway epithelial cell-specific consensomes for SARS1, SARS2 and MERS and computed intersections between their HCTs and the EMT signature. We found that significant intersection of the EMT signature with the CoV HCTs remained specific to SARS2 (*q*-values: SARS1 & MERS, 2e-1; SARS2, 1e-8) in the lung epithelium-specific CoV consensomes.

We next retrieved the canonical EMT genes in the SARS2 HCTs and compared their percentile rankings with the other CoV consensomes. Although some EMT genes, such as *CXCL2* and *IRF9*, had elevated rankings across all four viral consensomes, the collective EMT gene signature had a significantly higher mean percentile value in the SARS2 consensome than in each of the other viral consensomes (Fig. 7b; SARS2 mean percentile = 97.5; SARS1 mean percentile = 86, *p* = 1e-5, t-test SARS2 v SARS1; MERS mean percentile = 63, *p* = 1e-9, t-test SARS2 v MERS; IAV mean percentile = 76, *p* = 2e-7, t-test SARS2 v IAV)). A column named “EMT” in figshare File F1^12^, sections 2 (SARS1), 3 (SARS2), 4 (MERS) and 5 (IAV) identifies the ranking of the EMT genes in each of the viral consensomes.

Given that EMT has been linked to ARDs^74^, we speculated that the evidence connecting EMT and SARS2 acquired through our analysis might be reflected in the relatively strong intersection between ARDs markers in SARS2 HCTs compared to other viral HCTs. To test this hypothesis we carried out a PubMed search to identify a set of 88 expression biomarkers of ARDs or its associated pathology, acute lung injury (ALI). A column named “ALI/ARDs” in figshare File F1^12^, sections 2 (SARS1), 3 (SARS2) 4 (MERS) and 5 (IAV) identifies the expression biomarker genes using the PubMed identifiers for the original studies in which they were identified. Consistent with our hypothesis, we observed appreciable intersections between this gene set and the HCTs of all four viruses (SARS1 odds ratio (OR) = 7, *q* = 5e-9; SARS2 OR = 10.4, *q* = 1e-9; MERS, OR = 4.2, *q* = 2e-5; IAV OR = 6.8; *q* = 9e-8) with a particularly strong intersection evident in the SARS2 HCTs.

Although EMT has been associated with infection by transmissible gastroenteritis virus^84^ and IAV^83^, this is to our knowledge the first evidence connecting CoV infection, and specifically SARS2 infection, to an EMT signature. Interestingly, lipotoxin A4 has been shown to attenuate lipopolysaccharide-induced lung injury by reducing EMT^85^. Moreover, several members of the group of SARS2-induced EMT genes have been associated with signature pulmonary comorbidities of CoV infection, including *ADAR*^86^, *CLDN1*^87^ and *SOD2*^88^. Of note in the context of these data is the fact that signaling through two SARS2 cellular receptors, ACE2/AT2 and CD147/basigin, has been linked to EMT in the context of organ fibrosis^89–91^. Finally, a recent preprint has described EMT-like transcriptional and metabolic changes in response to SARS2 infection^92^. Collectively, our data indicate that EMT warrants further investigation as a SARS2-specific pathological mechanism.

### Hypothesis generation use case 4: SARS2 repression of E2F family HCTs encoding cell cycle regulators

**Fig. 8:**
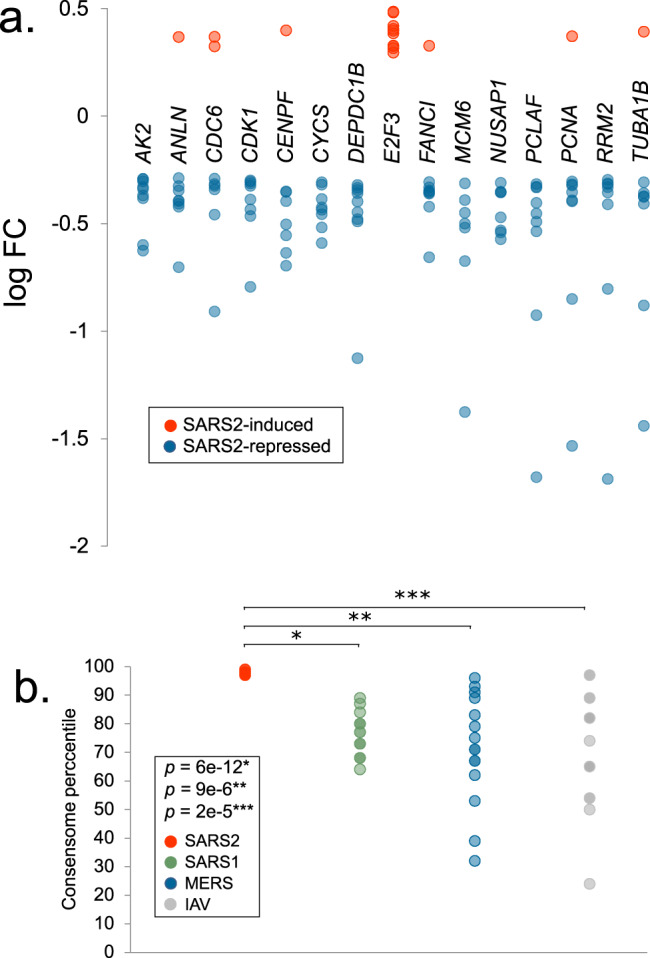
Hypothesis generation use case 4: efficient SARS2 repression of E2F family HCTs encoding key cell cycle regulators. **(a)** Relative abundance of E2F HCT cell cycle regulators in response to SARS2 infection. **(b**) Comparison of SARS2, SARS1, MERS and IAV consensome percentiles of the E2F HCT cell cycle regulators. Indicated are the results of the two-tailed two sample t-test assuming equal variance comparing the percentile rankings of the SARS2 EMT HCTs across the four viral consensomes.

Aside from EPAS1 and SNAI2, the only other transcription factors with significant HCT intersections that were specific to the SARS2 HCTs were the E2F/FOX class members E2F1 (*q*-values: SARS1, 5e-1; SARS2, 1e-2; MERS, 4e-1), E2F3 (*q*-values: SARS1, 6e-1; SARS2, 5e-2; MERS, 7e-1), E2F4 (*q*-values: SARS1, 1; SARS2, 9e-3; MERS, 1) and TFDP1/Dp-1 (*q*-values: SARS1, 1; SARS2, 3e-4; MERS, 1; Fig. 3, bold symbols; figshare File F1^12^, section 7). These factors play well-documented interdependent roles in the promotion (E2F1, E2F3, TFDP1) and repression (E2F4) of cell cycle genes^93,94^. Moreover, E2F family members are targets of signaling through EGFRs^95^ and CDK6^96^, both of whose HCTs had SARS2 HCT intersections that were stronger those of the other CoVs (EGFRs: q-values: SARS1, 4e-21; SARS2, 3e-48; MERS, 1e-35; CDK6: q-values: SARS1, 1e-7; SARS2, 8e-8; MERS, 2e-4); Figs. 2 and 4). Based on these data, we speculated that SARS2 infection might impact the expression of E2F-regulated cell cycle genes more efficiently than other CoVs. To investigate this we retrieved a set of SARS2 HCTs that were also HCTs for at least three of E2F1, E2F3, E2F4 and TFDP1 (figshare File F1^12^, section 3, columns E2F1, E2F3, E2F4, TFDP1 & 95th 3/4). Consistent with the role of E2F/Dp-1 nodes in the regulation of the cell cycle, many of these genes – notably *CDK1*, *PCNA*, *CDC6*, *CENPF* and *NUSAP1* – are critical positive regulators of DNA replication and cell cycle progression^97–101^ and are known to be transcriptionally induced by E2Fs^102–105^. Strikingly, with the exception of *E2F3*, all were consistently repressed in response to SARS2 infection (Fig. 8a). To gain insight into the relative efficiency with which the four viruses impacted expression of the E2F/Dp-1 HCT signature, we compared their mean percentile values across the viral consensomes. Consistent with efficient repression of the E2F/Dp-1 HCTs by SARS2 infection relative to other viruses, their mean percentile ranking was appreciably higher in the SARS2 consensome (97^th^ percentile) than in the SARS1 (76^th^ percentile; *p* = 6e-12, t-test SARS2 v SARS1), MERS (71.2 percentile; *p* = 9e-6, t-test SARS2 v MERS) and IAV (71.2 percentile; *p* = 2e-5, t-test SARS2 v IAV) consensomes (Fig. 8b). Although manipulation of the host cell cycle and evasion of detection through deregulation of cell cycle checkpoints has been described for other viruses^106–108^, this represents the first evidence for the profound impact of SARS2 infection on host cell cycle regulatory genes, potentially through disruption of E2F mediated signaling pathways. The SARS2 infection-mediated induction of *E2F3* (Fig. 8a) may represent a compensatory response to transcriptional repression of other E2F family members, as has been previously observed for this family in other contexts^109,110^. Consistent with our prediction in this use case, while this paper was in revision, a study appeared showing that infection by SARS2 results in cell cycle arrest^111^. Our results represent evidence that efficient modulation by SARS2 of E2F signaling, resulting in repression of cell cycle regulatory genes, may contribute to its unique pathological impact.

### Visualization of the CoV transcriptional regulatory networks in the Signaling Pathways Project knowledgebase and Network Data Exchange repository

To enable researchers to routinely generate mechanistic hypotheses around the interface between CoV infection human cell signaling, we next made the consensomes and accompanying HCT intersection analyses freely available to the research community in the SPP knowledgebase and the Network Data Exchange (NDEx) repository. Table 1 contains digital object identifier (DOI)-driven links to the consensome networks in SPP and NDEx, and to the virus-node and virus-node family HCT intersection networks in NDEx.

**Fig. 9:**
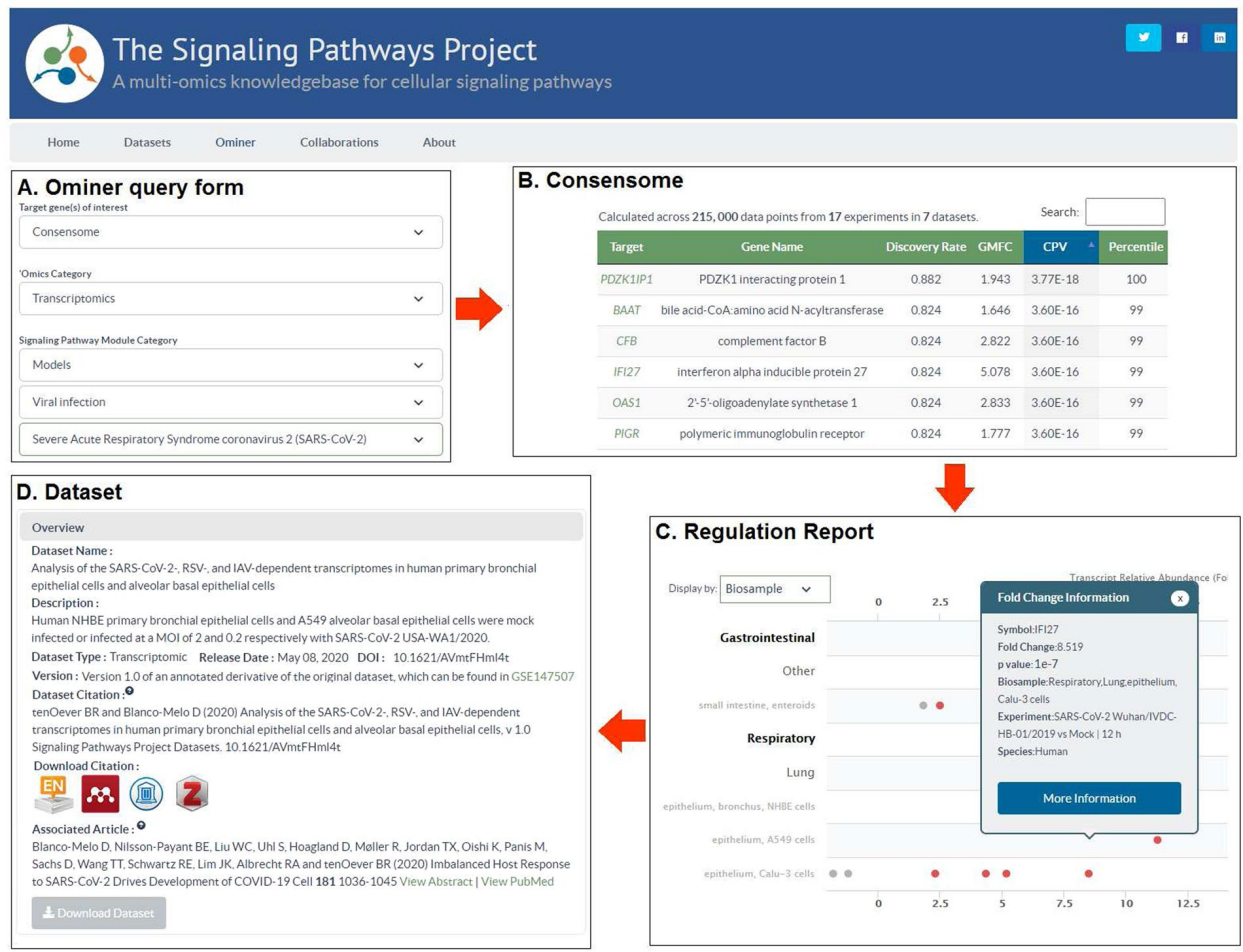
Mining of CoV consensomes and underlying data points in the SPP knowledgebase. (**A**) The Ominer query form can be configured as shown to access the CoV infection consensomes. In the example shown, the user wishes to view the SARS2 consensome. (**B**) Consensomes are displayed in a tabular format. Target transcript symbols in the consensomes link to SPP transcriptomic Regulation Reports (**C**) Regulation Reports for consensome transcripts are filtered to show only data points that contributed to their consensome ranking. Clicking on a data point opens a Fold Change Information window that links to the SPP curated version of the original archived dataset (**D**). Like all SPP datasets, CoV infection datasets are comprehensively aligned with FAIR data best practice and feature human-readable names and descriptions, a DOI, one-click addition to citation managers, and machine-readable downloadable data files. For a walk-through of CoV consensome data mining options in SPP, please refer to the accompanying YouTube video (http://tiny.cc/2i56rz).

We have previously described the SPP biocuration pipeline, database and web application interface^10^. Figure 9 shows the strategy for consensome data mining on the SPP website. The individual CoV consensomes can be accessed by configuring the SPP Ominer query form as shown, in this example for the SARS2 consensome (Fig. 9A). Figure 9B shows the layout of the consensomes, showing gene symbol, name, percentile ranking and other essential information. Genes in the 90^th^ percentile of each consensome are accessible via the user interface, with the full consensomes available for download in a tab delimited text file. Target gene symbols in the consensome link to the SPP Regulation Report, filtered to show only experimental data points that contributed to that specific consensome (Fig. 9C). This view gives insights into the influence of tissue and cell type context on the regulatory relationship. These filtered reports can be readily converted to default Reports that show evidence for regulation of a specific gene by other signaling pathway nodes. As previously described, pop-up windows in the Report provide experimental details, in addition to links to the parent dataset (Fig. 9D), curated accordingly to our previously described protocol^10^. Per FAIR data best practice, CoV infection datasets – like all SPP datasets – are associated with detailed descriptions, assigned a DOI, and linked to the associated article to place the dataset in its original experimental context (Fig. 9D). The full list of datasets is available for browsing in the SPP Dataset listing (https://www.signalingpathways.org/index.jsf).

**Fig. 10:**
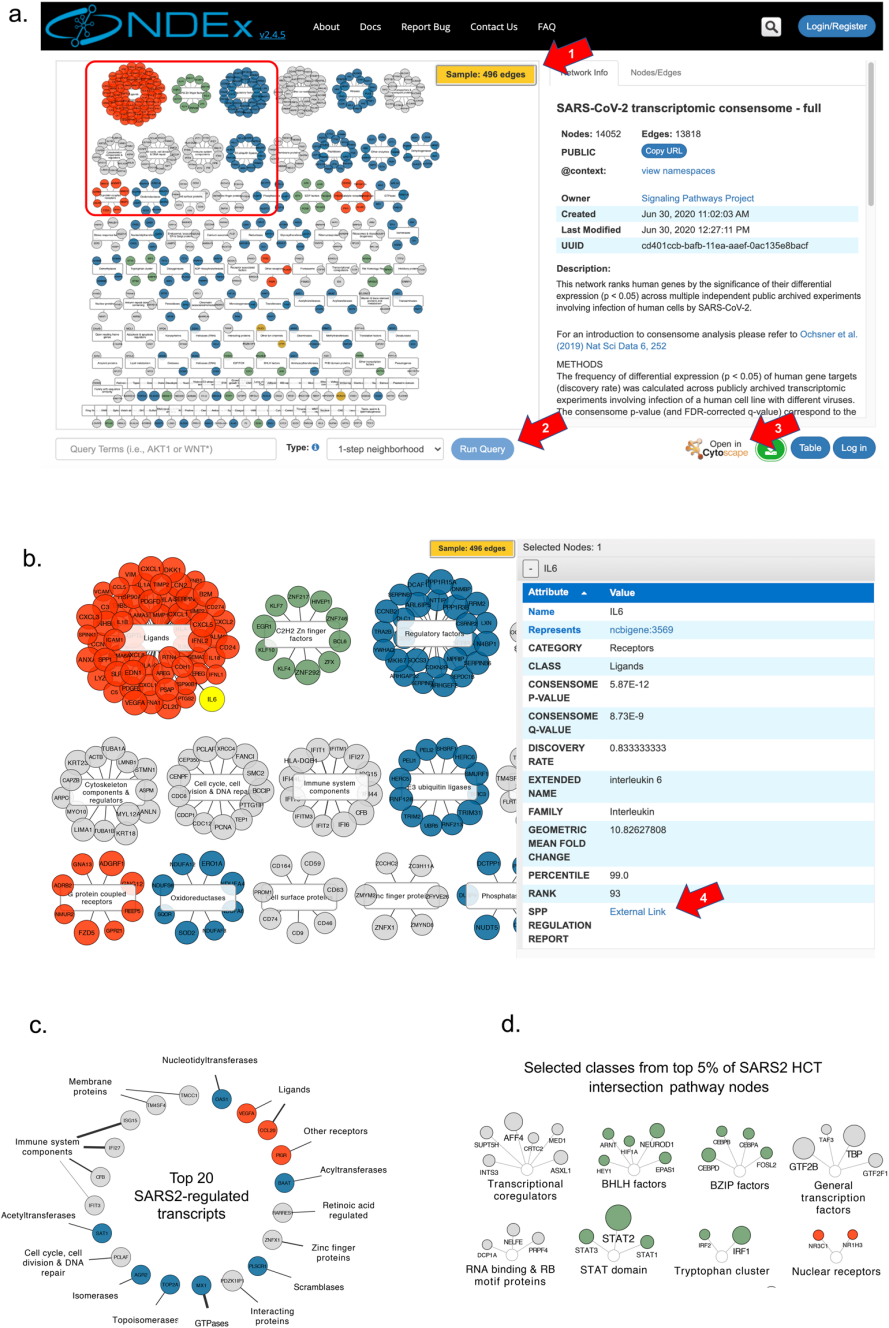
Visualization of viral consensomes and HCT intersection networks in the NDEx repository. In all panels, transcripts (consensome networks; panels a–c) and nodes (HCT intersection network; panel d) are color-coded according to their category as follows: receptors (orange), enzymes (blue), transcription factors (green), ion channels (mustard) and co-nodes (grey). Additional contextual information is available in the description of each network on the NDEx site. Red arrows are explained in the text. (**a**) Sample view of SARS2 consensome showing top 5% of transcripts. White rectangles represent classes to which transcripts have been mapped in the SPP biocuration pipeline^10^. Orange inset refers to the zoomed-in view in panel (**b**). The IL6 transcript is highlighted to show the contextual information available in the info table to the right. (**c**) Top 20 ranked transcripts in the SARS2 consensome. Edge thickness is proportional to the GMFC. (**d**) Selected classes represented in the top 5% of nodes in the SARS2 ChIP-Seq HCT intersection network. Node circle size is inversely proportional to the intersection q-value.

The NDEx repository facilitates collaborative publication of biological networks, as well as visualization of these networks in web or desktop versions of the popular and intuitive Cytoscape platform^112–114^. Figure 10 shows examples of consensome and HCT intersection network visualizations within the NDEx user interface, in which transcripts or nodes, respectively, are organized using SPP’s Category-Class-Family classification^10^. For ease of viewing, the initial rendering of the full SARS2 (Fig. 10a) and other consensome networks shows a sample (Fig. 10a, red arrow 1) containing only the top 5% of regulated transcripts; the full data can be explored using the “Neighborhood Query” feature available at the bottom of the page (red arrow 2). The integration in NDEx of the popular Cytoscape desktop application enables any network to be seamlessly be imported in Cytoscape for additional analysis (red arrow 3). Zooming in on a subset of the SARS2 consensome (orange box) affords an appreciation of the diversity of molecular classes that are transcriptionally regulated in response to SARS2 infection (Fig. 10b). Transcript size is proportional to rank percentile, and edge weight is proportional to the transcript geometric mean fold change (GMFC) value. Selecting a transcript allows the associated consensome data, such as rank, GMFC and family, to be examined in detail using the info table (Fig. 10b, right panel). Highlighted to exemplify this feature is IL6, an inflammatory ligand that has been previously linked to SARS2 infection-related pathology^8,115^. Consensome GMFCs are signless with respect to direction of regulation^10^. Researchers can therefore follow the SPP link in the info table (Fig. 10b, red arrow 4) to view the individual underlying viral infection data points on the SPP site (Fig. 9C shows the example for *IFI27*).

A network of the top 20 ranked transcripts in the SARS2 consensome (Fig. 10c) includes genes with known (*OAS1*, *MX1*^116^) and previously uncharacterized (*PDZKIP1*, *SAT1*, *TM4SF4*) transcriptional responses to SARS2 infection. Finally, to afford insight into pathway nodes whose gain or loss of function contributes to SARS2 infection-induced signaling, Fig. 10d shows the top 5% ranked nodes in the SARS2 node HCT ChIP-Seq intersection network (see figshare File F1^12^, section 7; see also Figs. 2 and 3 and accompanying discussion above). In this, as with all HCT intersection networks, node size is proportional to the *q*-value, such that the larger the circle, the lower the *q*-value, and the higher the confidence that a particular node or node family is involved in the transcriptional response to viral infection.

The NDEx interface leverages the SPP classification system to provide visual insights into the impact of CoV infection on human cell signaling that are not readily appreciated in the current SPP interface. For example, it is readily apparent from the NDEx SARS2 consensome network (Fig. 10c; Table 1) that the single largest class of SARS2 HCTs encodes immunomodulatory ligands (OR = 4.6, *p* = 3.8 e-24, hypergeometric test), many of which are members of the cytokine and chemokine superfamilies. In contrast, although still overabundant (OR = 1.58, p = 6.8e-4, hypergeometric test), inflammatory ligands comprise a considerably smaller proportion of the SARS1 HCTs (Table 1). These data represent evidence that compared to SARS1, SARS2 infection may be relatively efficient in modulating a transcriptional inflammatory response in host cells.

## Discussion

An effective research community response to the impact of CoV infection on human health demands systematic exploration of the transcriptional interface between CoV infection and human cell signaling systems. It also demands routine access to computational analysis of existing datasets that is unhindered either by paywalls or by lack of the informatics training required to manipulate archived datasets in their unprocessed state. Moreover, the substantial logistical obstacles to high containment laboratory certification emphasize the need for fullest possible access to, and re-usability of, existing CoV infection datasets to focus and refine hypotheses prior to carrying out *in vivo* CoV infection experiments. Meta-analysis of existing datasets represents a powerful approach to establishing consensus transcriptional signatures – consensomes – which identify those human genes whose expression is most consistently and reproducibly impacted by CoV infection. Moreover, integrating these consensus transcriptional signatures with existing consensomes for cellular signaling pathway nodes can illuminate transcriptional convergence between CoV infection and human cell signaling nodes.

To this end, we generated a set of CoV infection consensomes that rank human genes by the reproducibility of their differential expression (*p* < 0.05) in response to infection of human cells by CoVs. Using HCT intersection analysis, we then computed the CoV consensomes against high confidence transcriptional signatures for a broad range of cellular signaling pathway nodes, affording investigators with a broad range of signaling interests an entrez into the study of CoV infection of human cells. Although other enrichment based pathway analysis tools exist^117^, HCT intersection analysis differs from these by computing against only genes that have the closest predicted regulatory relationships with upstream pathway nodes (i.e. HCTs). The use cases described here represent illustrative examples of the types of HCT-based analyses that users are empowered to carry out to illuminate principles of CoV infection signaling.

Previous network analyses across independent viral infection transcriptomic datasets, although valuable, have been limited to stand-alone studies^118,119^. Here, to enable access to the CoV consensomes and their >3,000,000 underlying data points by the broadest possible audience, we have integrated them into the SPP knowledgebase and NDEx repository to create a unique, federated environment for generating hypotheses around the impact of CoV infection on human cell signaling. NDEx provides users with the familiar look and feel of Cytoscape to reduce barriers of accessibility, and provides for intuitive click-and-drag data mining strategies. Incorporation of the CoV data points into SPP places them in the context of millions more existing SPP data points documenting transcriptional regulatory relationships between human pathway nodes and their transcriptional targets. In doing so, we provide users with evidence for signaling nodes whose gain or loss of function in response to CoV infection gives rise to these transcriptional patterns. The transcriptional impact of viral infection is known to be an amalgam of host antiviral responses and co-option by viruses of the host signaling machinery in furtherance of its life cycle. It is hoped that dissection of these two distinct modalities in the context of CoV infection will be facilitated by the availability of the CoV consensomes in the SPP and NDEx knowledgebases.

The CoV consensomes have a number of limitations. Primarily, since they are predicated specifically on transcriptional regulatory technologies, they will assign low rankings to transcripts that may not be transcriptionally responsive to CoV infection, but whose encoded proteins nevertheless play a role in the cellular response. For example, *MASP2*, which encodes an important node in the response to CoV infection^120^, has either a very low consensome ranking (SARS1, MERS and IAV), or is absent entirely (SARS2), indicating that it is transcriptionally unresponsive to viral infection and likely activated at the protein level in response to upstream signals. This and similar instances therefore represent “false negatives” in the context of the impact of CoV infection on human cells. Another limitation of the transcriptional focus of the datasets is the absence of information on specific protein interactions and post-translational modifications, either viral-human or human-human, that give rise to the observed transcriptional responses. Although these can be inferred to some extent, integration of existing^34,70,111^ and future proteomic and kinomic datasets will facilitate modeling of the specific signal transduction events giving rise to the downstream transcriptional responses. Finally, although detailed metadata are readily available on the underlying data points, the consensomes do not directly reflect the impact of variables such as tissue context or duration of infection on differential gene expression. As additional suitable archived datasets become available, we will be better positioned to generate more specific consensomes of this nature.

The human CoV and IAV consensomes and their underlying datasets are intended as “living” resources in SPP and NDEx that will be updated and versioned with appropriate datasets as resources permit. This will be particularly important in the case of SARS2, given the expanded budget that worldwide funding agencies are likely to allocate to research into the impact of this virus on human health. Incorporation of future datasets will allow for clarification of observations that are intriguing, but whose significance is currently unclear, such as the intersection between the CoV HCTs and those of the telomerase catalytic subunit (figshare File F2^12^), as well as the enrichment of EMT genes among those with elevated rankings in the SARS2 consensome (Fig. 7). Although they are currently available on the SPP website, distribution of the CoV consensome data points via the SPP RESTful API^10^ will be essential for the research community to fully capitalize on this work. For example, several co-morbidities of SARS2 infection, including renal and gastrointestinal disorders, are within the mission of the National Institute of Diabetes, Digestive and Kidney Diseases. In an ongoing collaboration with the NIDDK Information Network (DKNET)^121^, the SPP API will make the CoV consensome data points available in a hypothesis generation environment that will enable users to model intersections of CoV infection-modulated host signaling with their own research areas of interest. We welcome feedback and suggestions from the research community for the future development of the CoV infection consensomes and HCT node intersection networks.

## Methods

Consistent with emerging NIH mandates on rigor and reproducibility, we have used the Research Resource Identifier (RRID) standard^122^ to identify key research resources of relevance to our study.

### Dataset biocuration

Datasets from Gene Expression Omnibus (RRID: SCR_005012) and Array Express (RRID: SCR_002964) were biocurated as previously described, with the incorporation of an additional classification of peptide ligands^123^ to supplement the existing mappings derived from the International Union of Pharmacology Guide To Pharmacology (RRID: SCR_013077).

### Dataset processing and consensome analysis

*Array data processing*. To process microarray expression data, we utilized the log2 summarized and normalized array feature expression intensities provided by the investigator and housed in GEO. These data are available in the corresponding “Series Matrix Files(s)”. The full set of summarized and normalized sample expression values were extracted and processed in the statistical program R. To calculate differential gene expression for investigator-defined experimental contrasts, we used the linear modeling functions from the Bioconductor limma analysis package^124^. Initially, a linear model was fitted to a group-means parameterization design matrix defining each experimental variable. Subsequently, we fitted a contrast matrix that recapitulated the sample contrasts of interest, in this case viral infection vs mock infection, producing fold-change and significance values for each array feature present on the array. The current BioConductor array annotation library was used for annotation of array identifiers. P values obtained from limma analysis were not corrected for multiple comparisons. *RNA-Seq data processing.* To process RNA-Seq expression data, we utilized the aligned, un-normalized, gene summarized read count data provided by the investigator and housed in GEO. These data are available in the corresponding “Supplementary file” section of the GEO record. The full set of raw aligned gene read count values were extracted and processed in the statistical program R using the limma^124^ and edgeR analysis^125^ packages. Read count values were initially filtered to remove genes with low read counts. Gene read count values were passed to downstream analysis if all replicate samples from at least one experimental condition had cpm >1. Sequence library normalization factors were calculated to apply scale normalization to the raw aligned read counts using the TMM normalization method implemented in the edgeR package followed by the voom function^126^ to convert the gene read count values to log2-cpm. The log2-cpm values were initially fit to a group-means parameterization design matrix defining each experimental variable. This was subsequently fit to a contrast design matrix that recapitulates the sample contrasts of interest, in this case viral infection vs mock infection, producing fold-change and significance values for each aligned sequenced gene. If necessary, the current BioConductor human organism annotation library was used for annotation of investigator-provided gene identifiers. P values obtained from limma analysis were not corrected for multiple comparisons.

Differential expression values were committed to the consensome analysis pipeline as previously described^10^. Briefly, the consensome algorithm surveys each experiment across all datasets and ranks genes according to the frequency with which they are significantly differentially expressed. For each transcript, we counted the number of experiments where the significance for differential expression was ≤0.05, and then generated the binomial probability, referred to as the consensome p-value (CPV), of observing that many or more nominally significant experiments out of the number of experiments in which the transcript was assayed, given a true probability of 0.05. Genes were ranked firstly by CPV, then by geometric mean fold change (GMFC). A more detailed description of the transcriptomic consensome algorithm is available in a previous publication^10^. The consensomes and underlying datasets were loaded into an Oracle 15c database and made available on the SPP user interface as previously described^10^.

### Statistical analysis

High confidence transcriptional target intersection analysis was performed using the Bioconductor GeneOverlap analysis package^18^ (RRID: SCR_018419) implemented in R. Briefly, given a whole set I of IDs and two sets A ∈ I and B ∈ I, and S = A ∩ B, GeneOverlap calculates the significance of obtaining S. The problem is formulated as a hypergeometric distribution or contingency table, which is solved by Fisher’s exact test. *p*-values were adjusted for multiple testing by using the method of Benjamini & Hochberg to control the false discovery rate as implemented with the p.adjust function in R, to generate q-values. The universe for the intersection was set at a conservative estimate of the total number of transcribed (protein and non protein-coding) genes in the human genome (25,000)^127^. R code for analyzing the intersection between an investigator gene set and CoV consensome HCTs has been deposited in the SPP Github account. A two tailed two sample t-test assuming equal variance was used to compare the mean percentile ranking of the EMT (12 degrees of freedom) and E2F (14 degrees of freedom) signatures in the MERS, SARS1, SARS2 and IAV consensomes using the PRISM software package v 7.0 (RRID: SCR_005375).

### Consensome generation

The procedure for generating transcriptomic consensomes has been previously described^10^. To generate the ChIP-Seq consensomes, we first retrieved processed gene lists from ChIP-Atlas^128^ (RRID: SCR_015511), in which human genes are ranked based upon their average MACS2 occupancy across all publically archived datasets in which a given pathway node is the IP antigen. Of the three stringency levels available (10, 5 and 1 kb from the transcription start site), we selected the most stringent (1 kb). According to SPP convention^10^, we then mapped the IP antigen to its pathway node category, class and family, and the experimental cell line to its appropriate biosample physiological system and organ. We then organized the ranked lists into percentiles to generate the node ChIP-Seq consensomes. The 95^th^ percentiles of all consensomes (HCTs, high confidence transcriptional targets) was used as the input for the HCT intersection analysis.

### SPP web application

The SPP knowledgebase (RRID: SCR_018412) is a gene-centric Java Enterprise Edition 6, web-based application around which other gene, mRNA, protein and BSM data from external databases such as NCBI are collected. After undergoing semiautomated processing and biocuration as described above, the data and annotations are stored in SPP’s Oracle 15c database. RESTful web services exposing SPP data, which are served to responsively designed views in the user interface, were created using a Flat UI Toolkit with a combination of JavaScript, D3.JS, AJAX, HTML5, and CSS3. JavaServer Faces and PrimeFaces are the primary technologies behind the user interface. SPP has been optimized for Firefox 24+, Chrome 30+, Safari 5.1.9+, and Internet Explorer 9+, with validations performed in BrowserStack and load testing in LoadUIWeb. XML describing each dataset and experiment is generated and submitted to CrossRef (RRID: SCR_003217) to mint DOIs^10^.

## Data Availability

Important note on data availability: this paper refers to the first versions of the consensomes and HCT intersection networks based on the datasets available at the time of publication. As additional CoV infection datasets are archived over time, we will make updated versions of the consensomes and HCT intersection analyses accessible in future releases. The entire set of experimental metadata is available in figshare File F1^12^, section 1. Consensome data points are in figshare File F1^12^, sections 2–5. **SPP** SPP MERS^129^, SARS1^130^, SARS2^131^ and IAV^132^ consensomes, their underlying data points and metadata, as well as original datasets, are freely accessible at https://ww.signalingpathways.org. Programmatic access to all underlying data points and their associated metadata are supported by a RESTful API at https://www.signalingpathways.org/docs/. All SPP datasets are biocurated versions of publically archived datasets, are formatted according to the recommendations of the FORCE11 Joint Declaration on Data Citation Principles, and are made available under a Creative Commons CC BY 4.0 license. The original datasets are available are linked to from the corresponding SPP datasets using the original repository accession identifiers. These identifiers are for transcriptomic datasets, the Gene Expression Omnibus (GEO) Series (GSE); and for cistromic/ChIP-Seq datasets, the NCBI Sequence Read Archive (SRA) study identifier (SRP). SPP consensomes are assigned DOIs as shown in Table 1. **NDEx** NDEx versions of consensomes (MERS^133^, SARS1^134^, SARS2^135^ and IAV^136^) and node family (MERS^137^, SARS1^138^, SARS2^139^ and IAV^140^) and node (MERS^141^, SARS1^142^, SARS2^143^ and IAV^144^) HCT intersection networks are freely available in the NDEx repository and assigned DOIs as shown in Table 1. NDEx is a recommended repository for Scientific Data, Springer Nature and the PLOS family of journals and is registered on FAIRsharing.org; for additional info and documentation, please visit http://ndexbio.org. The official SPP account in NDEx is available at: https://bit.ly/30nN129.

## Code availability

SPP source code is available in the SPP GitHub account under a Creative Commons CC BY 4.0 license at https://github.com/signaling-pathways-project.
